# Supplementary figures and images for: Exogenous application of nitric oxide donors regulates short-term flooding stress in soybean
Source: PeerJ. 2019 Oct 8;7:e7741. doi: 10.7717/peerj.7741 (PMC6788439; doi:10.7717/peerj.7741)

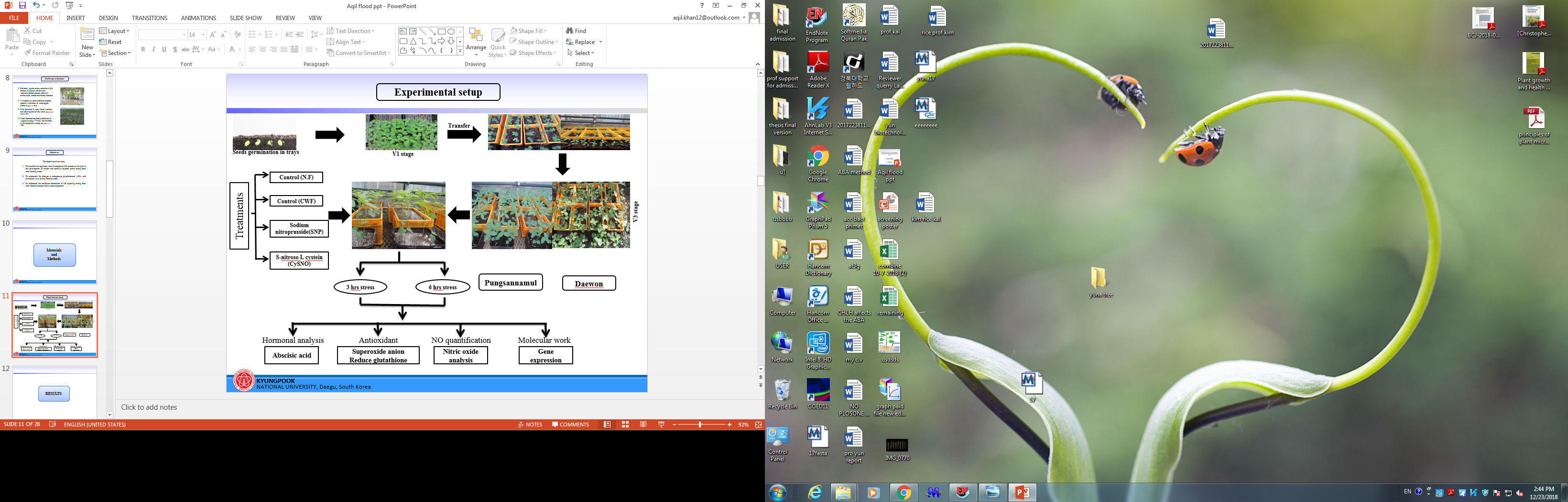

Supplement: Supplemental Information 3 [file peerj-07-7741-s003.docx]
